# Supplementary material for: Molecular Basis of Selective Cytokine Signaling Inhibition by Antibodies Targeting a Shared Receptor
Source: Front Immunol. 2021 Dec 24;12:779100. doi: 10.3389/fimmu.2021.779100 (PMC8740070; doi:10.3389/fimmu.2021.779100)
Supplement: Supplementary file 1 [file DataSheet_1.pdf]

Supplemental Figure 1: Deglycosylation of IL-1RAcP by Endoglycosidase A and IL-1/IL-33 Cell Signaling Inhibition (A) Lane 1: Bio-Rad unstained protein ladder. Lane 2: Endoglycosidase A (EndoA). Lane 3: Glycosylated IL-1RAcP. Lane 4: Deglycosylated IL-1RAcP. **(B)** Table containing the IC_{50} and R-square values for IL-1 and IL-33 cell signaling assays.

Supplemental Figure 2: Deuterium uptake of IL-1RAcP in apo form and antibody bound form by HDX-MS. (A) Deuterium uptake graphs of CAN03 bound (red), CAN04 bound (blue), and Apo IL-1RAcP (black). This stretch of residues was used in figure 3A for our HDX-MS analysis of CAN03 binding to IL-1RAcP. **(B)** Deuterium uptake graphs of CAN03 bound (red), CAN04 bound (blue), and Apo IL-1RAcP (black). This stretch of residues was used in figure 3B for our HDX-MS analysis of CAN04 binding to IL-1RAcP. **(C)** IL-1RAcP HDX-MS peptide coverage on IL-1RAcP, wherein coverage is colored green and lack of coverage is colored dark grey. **(D)** Peptide coverage map of IL-1RAcP, wherein coverage was 65.2% and redundancy was 2.96.

Supplemental Table 2: PISA analysis of IL-1 and IL-33 Signaling Complexes (A) Table of PISA interface of chain C (IL-1RAcP) to chain A (IL-1β) from the crystal structure of the IL-1β signaling complex (pdb: 4DEP). **(B)** Table of PISA interface of chain C (IL-1RAcP) to chain B (IL-1RI) from the crystal structure of the IL-1β signaling complex (pdb: 4DEP). **(C)** Table of PISA interface of chain C (IL-1RAcP) to chain A (IL-33) from the crystal structure of the IL-33 signaling complex (pdb: 5VI4). **(D)** Table of PISA interface of chain C (IL-1RAcP) to chain B (ST2) from the crystal structure of the IL-33 signaling complex (pdb: 5VI4).

Supplemental Figure 3: Sensorgrams from corresponding SPR experiments for Extensive Alanine Scan (A) Sensorgrams of IL-1RAcP alanine scan (Fc-fused IL-1RAcP with alanine mutations) and CAN03 as the analyte with Chi^2 values of fit. **(B)** Sensorgrams of IL-1RAcP alanine scan (Fc-fused IL-1RAcP with alanine mutations) and CAN04 as the analyte with Chi^2 values of fit.

Supplemental Table 3: Kinetic data from Extensive Alanine Scan (A) k_a, k_d, and K_D values of alanine scan SPR experiment, with CAN03 as the analyte. **(B)** k_a, k_d, and K_D values of alanine scan SPR experiment, with CAN04 as the analyte.

Supplemental Figure 4: CAN03 and CAN04 combination IL-1β Cell Signaling Inhibition Assay (A) A cell signaling assay containing CAN03 (red), CAN04 (blue), and a mix of CAN03/CAN04 (purple) to accompany Figure 7.

A

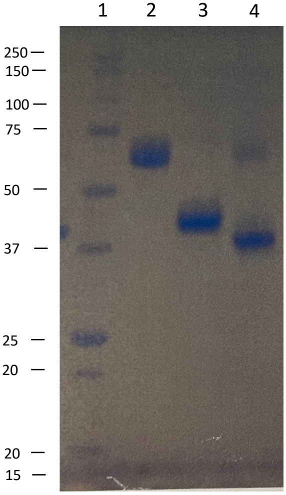

B

Antagonist	IC_{50} of antagonists for IL-1 Signaling Inhibition	R-Square of IL-1 Non-linear fit	IC_{50} of antagonists for IL-33 Signaling Inhibition	R-Square of IL-33 Non-linear fit
Interleukin-1 Receptor Antagonist (IL-1Ra)	$1.64 * 10^{-10}$	0.98	-	-
Soluble ST2	-	-	$1.16 * 10^{-10}$	0.97
CAN03	$4.37 * 10^{-9}$	0.88	$2.20 * 10^{-8}$	0.90
CAN04	$4.87 * 10^{-11}$	0.98	$7.82 * 10^{-10}$	0.90

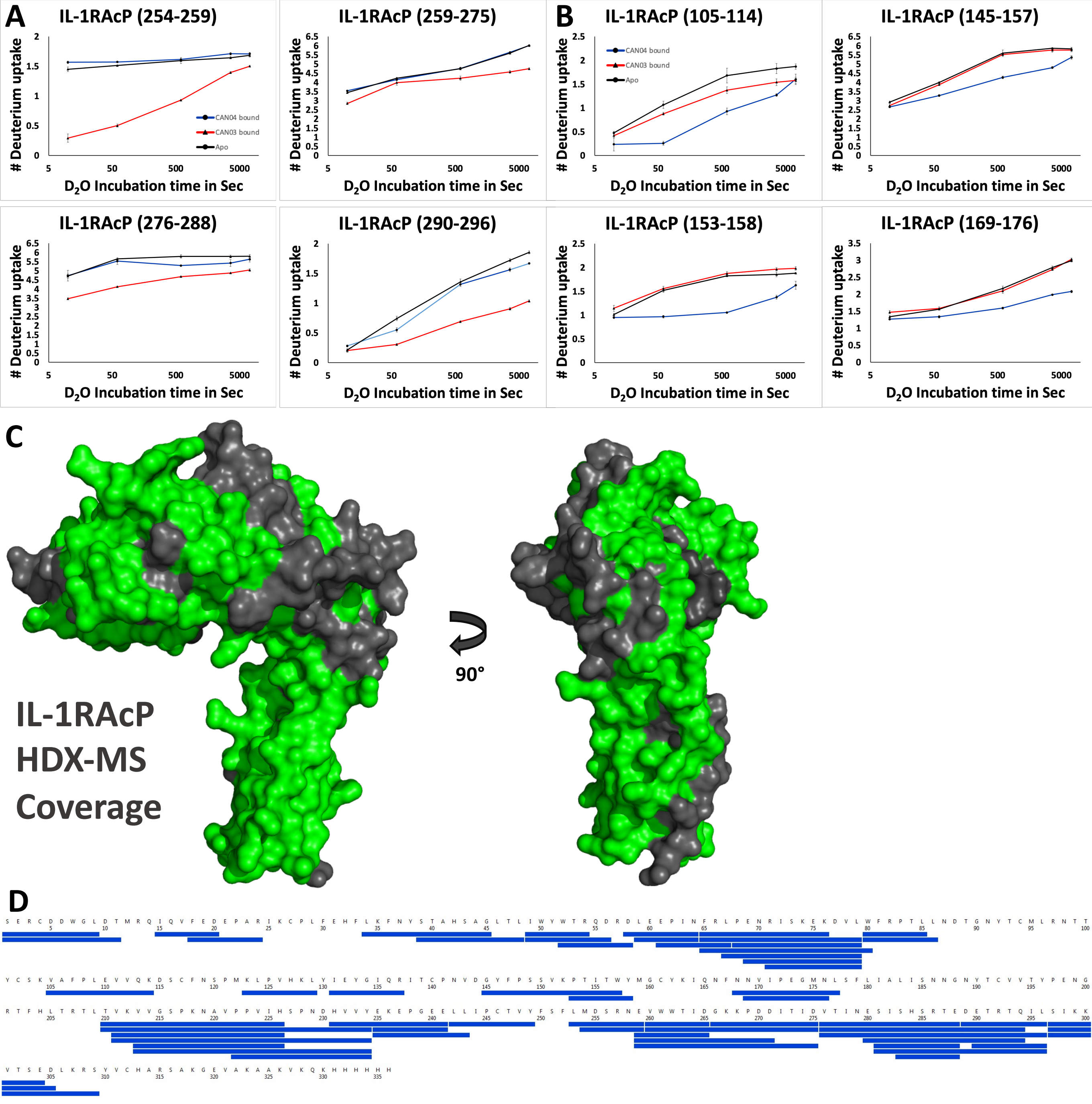
A
IL-1RAcP (254-259)
IL-1RAcP (259-275)
B
IL-1RAcP (105-114)
IL-1RAcP (145-157)
IL-1RAcP (276-288)
IL-1RAcP (290-296)
IL-1RAcP (153-158)
IL-1RAcP (169-176)
Deuterium uptake
D2O Incubation time in Sec
5 50 500 5000
CAN04 bound
CAN03 bound
Apo
C
90°
IL-1RAcP HDX-MS Coverage
D
S E R C D D W G L D T M R Q I Q V F E D E P A R I K C P L F E H F L K F N Y S T A H S A G L T L I W Y W T R Q D R D L E E P I N F R L P E N R I S K E K D V L W F R P T L L N D T G N Y T C M L R N T T
5 10 15 20 25 30 35 40 45 50 55 60 65 70 75 80 85 90 95 100
Y C S K V A F P L E V V Q K D S C F N S P M K L P V H K L Y I E Y G I Q R I T C P N V D G Y F P S S V K P T I T W Y M G C Y K I Q N F N N V I P E G M N L S F L I A L I S N N G N Y T C V V T Y P E N G
105 110 115 120 125 130 135 140 145 150 155 160 165 170 175 180 185 190 195 200
R T F H L T R T L T V K V V G S P K N A V P P V I H S P N D H V V Y E K E P G E E L L I P C T V Y F S F L M D S R N E V W W T I D G K K P D D I T I D V T I N E S I S H S R T E D E T R T Q I L S I K K
205 210 215 220 225 230 235 240 245 250 255 260 265 270 275 280 285 290 295 300
V T S E D L K R S Y V C H A R S A K G E V A K A A K V K Q K H H H H H H
305 310 315 320 325 330 335

A PISA Analysis: IL-1β interface on IL-1RAcP

<table>
<tr><th>Region</th><th>##</th><th>Structure 1</th><th>HSDC</th><th>ASA</th><th>BSA</th><th>ΔiG</th></tr>
<tr><td>Domain 2</td><td>129</td><td>C:ILE 131</td><td></td><td>35.1</td><td>29.76 |||||||||</td><td>0.48</td></tr>
<tr><td>Domain 2</td><td>130</td><td>C:GLU 132</td><td>S</td><td>105.5</td><td>51.52 |||||</td><td>-0.25</td></tr>
<tr><td>Domain 2</td><td>157</td><td>C:MET 159</td><td></td><td>51.9</td><td>23.85 |||||</td><td>0.85</td></tr>
<tr><td>Domain 2</td><td>160</td><td>C:TYR 162</td><td></td><td>163.9</td><td>15.49 |</td><td>0.25</td></tr>
<tr><td>Domain 2</td><td>163</td><td>C:GLN 165</td><td>H</td><td>66.6</td><td>14.10 |||</td><td>-0.07</td></tr>
<tr><td>Domain 2</td><td>164</td><td>C:ASN 166</td><td>H</td><td>155.7</td><td>52.41 ||||</td><td>-0.29</td></tr>
<tr><td>Domain 2</td><td>165</td><td>C:PHE 167</td><td></td><td>65.6</td><td>43.42 |||||||</td><td>0.56</td></tr>
<tr><td>Domain 2</td><td>166</td><td>C:ASN 168</td><td>H</td><td>140.2</td><td>113.28 |||||||||</td><td>0.02</td></tr>
<tr><td>Domain 2</td><td>167</td><td>C:ASN 169</td><td></td><td>18.8</td><td>0.61 |</td><td>-0.01</td></tr>
<tr><td>Domain 2</td><td>168</td><td>C:VAL 170</td><td></td><td>3.99</td><td>2.68 |||||||</td><td>0.04</td></tr>
<tr><td>Domain 2</td><td>179</td><td>C:ILE 181</td><td></td><td>90.6</td><td>1.49 |</td><td>0.02</td></tr>
<tr><td>Domain 2</td><td>181</td><td>C:LEU 183</td><td></td><td>79.0</td><td>64.84 |||||||||</td><td>1.04</td></tr>
<tr><td>Domain 2</td><td>182</td><td>C:ILE 184</td><td></td><td>75.5</td><td>59.26 ||||||||</td><td>0.82</td></tr>
<tr><td>Domain 2</td><td>183</td><td>C:SER 185</td><td></td><td>83.1</td><td>79.12 ||||||||||</td><td>0.71</td></tr>
<tr><td>Domain 2</td><td>184</td><td>C:ASN 186</td><td></td><td>8.18</td><td>8.18 |||||||||||</td><td>-0.09</td></tr>
<tr><td>Domain 2</td><td>188</td><td>C:TYR 190</td><td></td><td>1.40</td><td>0.49 ||||</td><td>-0.01</td></tr>
<tr><td>Linker</td><td>217</td><td>C:ASN 219</td><td></td><td>105.8</td><td>14.86 ||</td><td>-0.17</td></tr>
<tr><td>Domain 3</td><td>260</td><td>C:ARG 286</td><td>HS</td><td>183.7</td><td>106.51 ||||||</td><td>-0.98</td></tr>
<tr><td>Domain 3</td><td>261</td><td>C:THR 287</td><td></td><td>29.4</td><td>16.16 ||||||</td><td>0.23</td></tr>
</table>

<table>
<tr><th>Region</th><th>Domain Location of Interaction</th></tr>
<tr><td>##</td><td>Line Number</td></tr>
<tr><td>Structure 1</td><td>Chain – Amino Acid - # in PDB file</td></tr>
<tr><td>HSDC</td><td>Residues making Hydrogen/Disulphide bond, Salt Bridge, or Covalent Link.</td></tr>
<tr><td>ASA</td><td>Accessible Surface Area, Å²</td></tr>
<tr><td>BSA</td><td>Buried Surface Area, Å²</td></tr>
<tr><td>ΔⁱG</td><td>Solvation energy effect, kcal/mol</td></tr>
<tr><td>||||</td><td>Buried area percentage, one bar per 10%</td></tr>
</table>

B

PISA Analysis: IL-1RI interface on IL-1RAcP

<table>
<tr><th>Region</th><th>##</th><th>Structure 1</th><th>HSDC</th><th>ASA</th><th>BSA</th><th>ΔiG</th></tr>
<tr><td>Domain 2</td><td>130</td><td>C:GLU 132</td><td></td><td>105.5</td><td>47.49 |||||</td><td>-0.49</td></tr>
<tr><td>Domain 2</td><td>131</td><td>C:TYR 133</td><td></td><td>76.9</td><td>3.29 |</td><td>0.05</td></tr>
<tr><td>Domain 2</td><td>132</td><td>C:GLY 134</td><td>H</td><td>37.9</td><td>36.15 ||||||||||</td><td>0.38</td></tr>
<tr><td>Domain 2</td><td>133</td><td>C:ILE 135</td><td></td><td>106.6</td><td>74.39 |||||||</td><td>1.14</td></tr>
<tr><td>Domain 2</td><td>135</td><td>C:ARG 137</td><td></td><td>125.1</td><td>4.96 |</td><td>-0.18</td></tr>
<tr><td>Domain 2</td><td>164</td><td>C:ASN 166</td><td></td><td>155.7</td><td>2.82 |</td><td>-0.05</td></tr>
<tr><td>Domain 2</td><td>165</td><td>C:PHE 167</td><td></td><td>65.6</td><td>4.54 |</td><td>-0.05</td></tr>
<tr><td>Domain 2</td><td>166</td><td>C:ASN 168</td><td>H</td><td>140.2</td><td>26.96 ||</td><td>-0.22</td></tr>
<tr><td>Domain 2</td><td>167</td><td>C:ASN 169</td><td></td><td>18.8</td><td>1.34 |</td><td>0.02</td></tr>
<tr><td>Domain 2</td><td>169</td><td>C:ILE 171</td><td></td><td>76.2</td><td>44.32 ||||||</td><td>0.71</td></tr>
<tr><td>Domain 2</td><td>171</td><td>C:GLU 173</td><td></td><td>74.9</td><td>9.46 ||</td><td>-0.16</td></tr>
<tr><td>Domain 2</td><td>178</td><td>C:LEU 180</td><td></td><td>63.7</td><td>57.32 |||||||||</td><td>0.92</td></tr>
<tr><td>Domain 2</td><td>179</td><td>C:ILE 181</td><td></td><td>90.6</td><td>63.33 |||||||</td><td>1.01</td></tr>
<tr><td>Domain 2</td><td>182</td><td>C:ILE 184</td><td></td><td>75.5</td><td>1.32 |</td><td>0.02</td></tr>
<tr><td>Linker</td><td>216</td><td>C:LYS 218</td><td>S</td><td>152.4</td><td>66.13 |||||</td><td>-0.97</td></tr>
<tr><td>Linker</td><td>217</td><td>C:ASN 219</td><td></td><td>105.8</td><td>55.59 ||||||</td><td>-0.25</td></tr>
<tr><td>Domain 3</td><td>224</td><td>C:HIS 226</td><td>S</td><td>169.8</td><td>82.39 |||||</td><td>0.39</td></tr>
<tr><td>Domain 3</td><td>227</td><td>C:PRO 245</td><td></td><td>79.3</td><td>7.85 |</td><td>0.13</td></tr>
<tr><td>Domain 3</td><td>229</td><td>C:THR 247</td><td></td><td>30.0</td><td>5.52 ||</td><td>0.09</td></tr>
<tr><td>Domain 3</td><td>231</td><td>C:TYR 249</td><td></td><td>45.4</td><td>22.25 |||||</td><td>0.36</td></tr>
<tr><td>Domain 3</td><td>259</td><td>C:SER 285</td><td></td><td>55.1</td><td>24.16 |||||</td><td>0.39</td></tr>
<tr><td>Domain 3</td><td>260</td><td>C:ARG 286</td><td></td><td>183.7</td><td>30.90 ||</td><td>0.28</td></tr>
<tr><td>Domain 3</td><td>261</td><td>C:THR 287</td><td></td><td>29.4</td><td>13.30 |||||</td><td>0.03</td></tr>
<tr><td>Domain 3</td><td>263</td><td>C:ASP 289</td><td></td><td>47.8</td><td>17.60 ||||</td><td>-0.21</td></tr>
<tr><td>Domain 3</td><td>265</td><td>C:THR 291</td><td>H</td><td>47.0</td><td>27.27 ||||||</td><td>0.11</td></tr>
</table>

<table>
<tr><td>Region</td><td>Domain Location of Interaction</td></tr>
<tr><td>##</td><td>Line Number</td></tr>
<tr><td>Structure 1</td><td>Chain – Amino Acid - # in PDB file</td></tr>
<tr><td>HSDC</td><td>Residues making Hydrogen/Disulphide bond, Salt Bridge, or Covalent Link.</td></tr>
<tr><td>ASA</td><td>Accessible Surface Area, Å²</td></tr>
<tr><td>BSA</td><td>Buried Surface Area, Å²</td></tr>
<tr><td>ΔⁱG</td><td>Solvation energy effect, kcal/mol</td></tr>
<tr><td>||||</td><td>Buried area percentage, one bar per 10%</td></tr>
</table>

C

PISA Analysis: IL-33 interface on IL-1RAcP

Region	##	Structure 1	HSDC	ASA	BSA	ΔiG
Domain 2	129	C:ILE 151		29.3	1.50 |	0.02
Domain 2	130	C:GLU 152	H	114.1	48.79 |||||	-0.45
Domain 2	157	C:LYS 179	HS	58.3	23.75 |||||	-0.18
Domain 2	164	C:ASP 186		83.2	20.12 |||	-0.23
Domain 2	165	C:PHE 187		72.3	48.31 |||||||	0.77
Domain 2	166	C:HIS 188	H	177.5	112.50 |||||||	-0.06
Domain 2	167	C:ASN 189		16.8	8.25 |||||	0.10
Domain 2	181	C:LEU 203		58.8	51.06 |||||||||	0.82
Domain 2	182	C:VAL 204		63.3	5.84 |	0.09
Domain 2	183	C:SER 205	H	87.0	60.08 |||||||	0.09
Domain 2	184	C:ASN 206		6.30	1.52 |||	-0.02
Domain 3	239	C:LYS 267		91.3	13.16 ||	-0.49
Domain 3	264	C:VAL 302		68.7	6.99 ||	-0.08
Domain 3	265	C:SER 303	H	65.8	49.12 ||||||||	0.24
Domain 3	266	C:TYR 304		132.7	17.50 ||	-0.20
Domain 3	267	C:SER 305		45.1	5.74 ||	0.09
Domain 3	268	C:SER 306		71.9	23.60 ||||	0.20

**Region**	Domain Location of Interaction
**##**	Line Number
**Structure 1**	Chain – Amino Acid - # in PDB file
**HSDC**	Residues making **H**ydrogen/**D**isulphide bond, **S**alt Bridge, or **C**ovalent Link.
**ASA**	Accessible Surface Area, Å²
**BSA**	Buried Surface Area, Å²
**ΔiG**	Solvation energy effect, kcal/mol
||||	Buried area percentage, one bar per 10%

D

PISA Analysis: ST2 interface on IL-1RAcP

Region	##	Structure 1	HSDC	ASA	BSA	ΔiG
Domain 2	129	C:ILE 151		29.3	5.90 |||	0.03
Domain 2	130	C:GLU 152		114.1	65.32 ||||||	-0.26
Domain 2	131	C:HIS 153		84.3	1.68 |	0.03
Domain 2	132	C:GLY 154		53.3	38.38 ||||||||	0.36
Domain 2	133	C:ILE 155	H	116.5	90.85 ||||||||	1.16
Domain 2	135	C:LYS 157		101.9	17.42 ||	0.06
Domain 2	164	C:ASP 186	HS	83.2	42.91 ||||||	-0.57
Domain 2	165	C:PHE 187	H	72.3	11.40 ||	-0.01
Domain 2	166	C:HIS 188	H	177.5	65.01 ||||	0.29
Domain 2	167	C:ASN 189		16.8	4.00 |||	0.06
Domain 2	168	C:VAL 190		0.12	0.12 |||||||||||	-0.00
Domain 2	169	C:LEU 191		62.8	21.25 ||||	0.34
Domain 2	171	C:GLU 193	HS	80.3	11.72 ||	-0.16
Domain 2	176	C:SER 198	H	1.54	1.54 ||||||||||	0.01
Domain 2	178	C:PHE 200		76.9	76.36 ||||||||||	1.15
Domain 2	179	C:ILE 201		101.3	87.43 |||||||||	1.40
Linker	214	C:SER 236		6.47	3.44 ||||||	-0.04
Linker	216	C:LYS 238	HS	171.7	63.95 ||||	-1.06
Linker	217	C:ASP 239	HS	92.5	71.14 ||||||||	-0.15
Domain 3	219	C:LEU 241		87.1	69.44 ||||||||	1.11
Domain 3	220	C:PRO 242		52.1	12.23 |||	0.20
Domain 3	221	C:PRO 243		5.26	1.35 |||	-0.02
Domain 3	222	C:GLN 244	H	68.8	67.95 ||||||||||	-0.52
Domain 3	223	C:ILE 245	H	35.5	35.56 ||||||||||	-0.21
Domain 3	224	C:TYR 246		116.7	113.59 ||||||||||	0.91
Domain 3	225	C:SER 247		26.8	9.37 ||||	0.15
Domain 3	226	C:PRO 248		1.72	1.22 ||||||||	-0.01
Domain 3	227	C:ASN 249	H	62.1	52.72 |||||||||	-0.11
Domain 3	228	C:ASP 250		83.6	16.04 ||	-0.11
Domain 3	229	C:ARG 251		227.9	119.57 ||||||	-0.89
Domain 3	230	C:VAL 252		53.0	30.94 ||||||	0.50
Domain 3	239	C:LYS 267		91.3	15.66 ||	-0.42
Domain 3	241	C:TYR 269		53.4	40.91 ||||||||	0.24
Domain 3	243	C:SER 271		5.37	1.10 |||	-0.01
Domain 3	298	C:ALA 341		23.8	9.37 ||||	0.15
Domain 3	299	C:GLU 342		121.9	10.68 |	-0.12
Domain 3	300	C:GLN 343	H	56.9	30.03 ||||||	-0.27

**Region**	Domain Location of Interaction
**##**	Line Number
**Structure 1**	Chain – Amino Acid - # in PDB file
**HSDC**	Residues making **H**ydrogen/**D**isulphide bond, **S**alt Bridge, or **C**ovalent Link.
**ASA**	Accessible Surface Area, Å²
**BSA**	Buried Surface Area, Å²
**Δ^{i}G**	Solvation energy effect, kcal/mol
| | | |	Buried area percentage, one bar per 10%

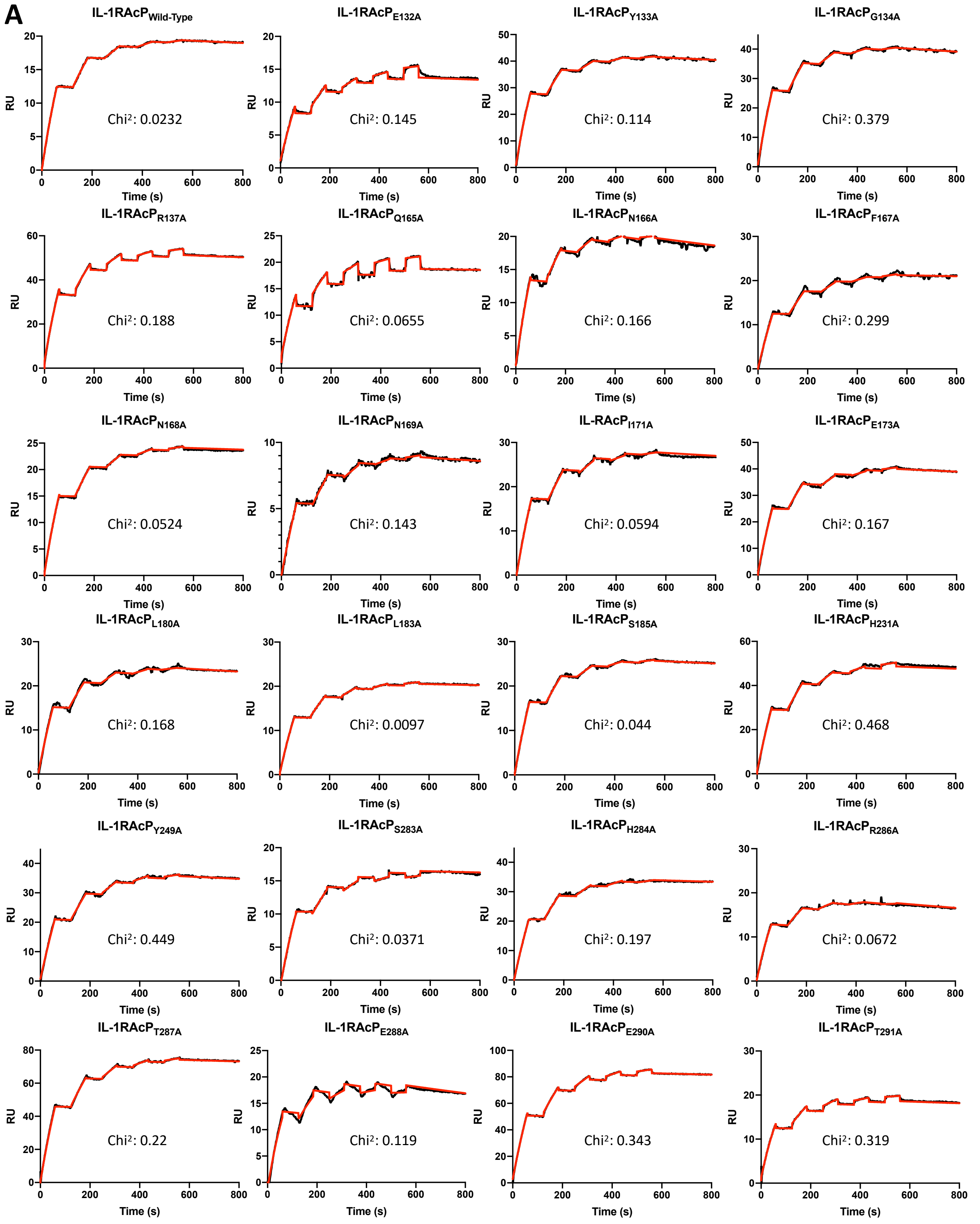

A
IL-1RAcP$_{Wild-Type}$
Chi²: 0.0232
IL-1RAcP$_{E132A}$
Chi²: 0.145
IL-1RAcP$_{Y133A}$
Chi²: 0.114
IL-1RAcP$_{G134A}$
Chi²: 0.379
IL-1RAcP$_{R137A}$
Chi²: 0.188
IL-1RAcP$_{Q165A}$
Chi²: 0.0655
IL-1RAcP$_{N166A}$
Chi²: 0.166
IL-1RAcP$_{F167A}$
Chi²: 0.299
IL-1RAcP$_{N168A}$
Chi²: 0.0524
IL-1RAcP$_{N169A}$
Chi²: 0.143
IL-RAcP$_{I171A}$
Chi²: 0.0594
IL-1RAcP$_{E173A}$
Chi²: 0.167
IL-1RAcP$_{L180A}$
Chi²: 0.168
IL-1RAcP$_{L183A}$
Chi²: 0.0097
IL-1RAcP$_{S185A}$
Chi²: 0.044
IL-1RAcP$_{H231A}$
Chi²: 0.468
IL-1RAcP$_{Y249A}$
Chi²: 0.449
IL-1RAcP$_{S283A}$
Chi²: 0.0371
IL-1RAcP$_{H284A}$
Chi²: 0.197
IL-1RAcP$_{R286A}$
Chi²: 0.0672
IL-1RAcP$_{T287A}$
Chi²: 0.22
IL-1RAcP$_{E288A}$
Chi²: 0.119
IL-1RAcP$_{E290A}$
Chi²: 0.343
IL-1RAcP$_{T291A}$
Chi²: 0.319
RU
Time (s)

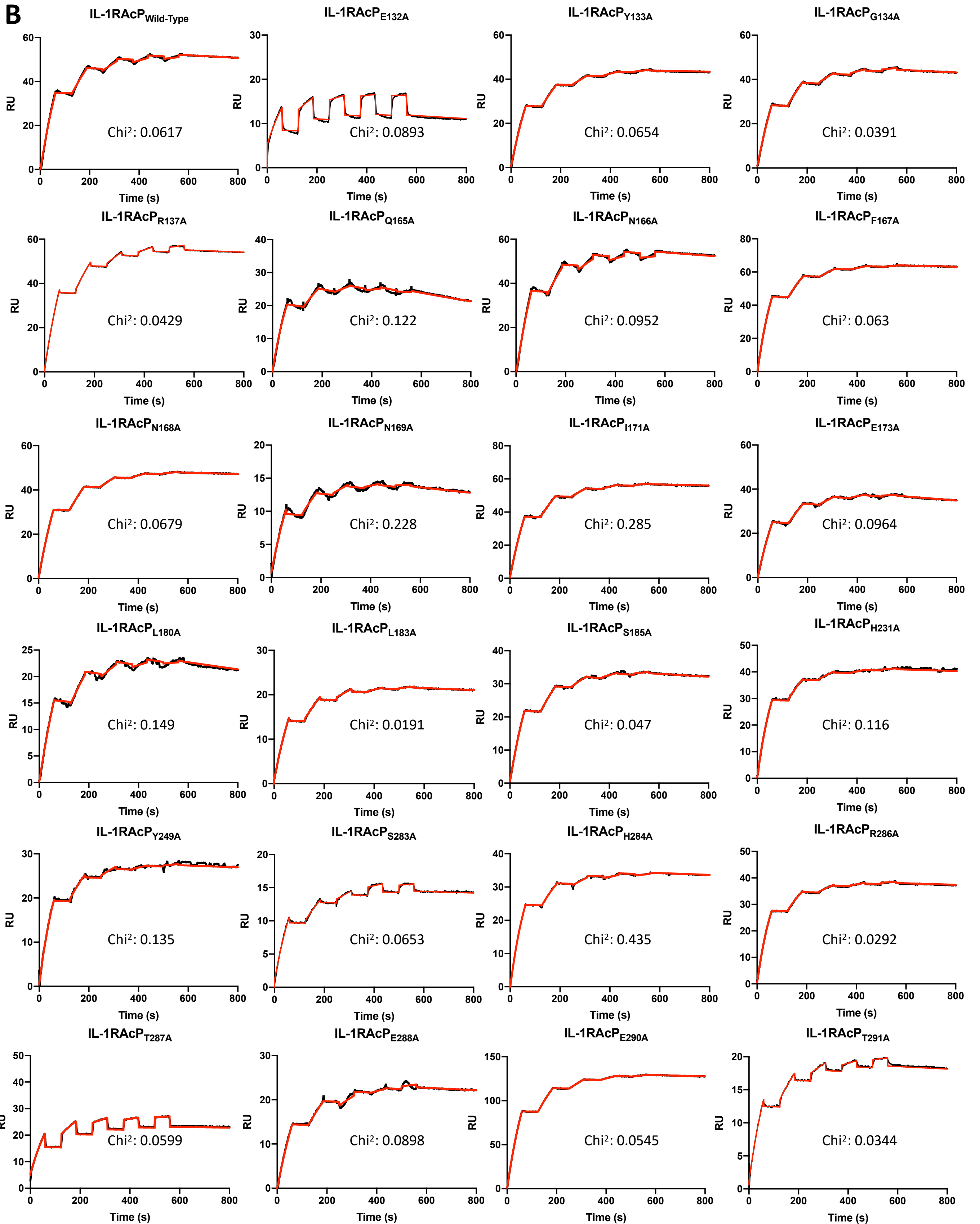
B
IL-1RAcP Wild-Type
Chi²: 0.0617
IL-1RAcP E132A
Chi²: 0.0893
IL-1RAcP Y133A
Chi²: 0.0654
IL-1RAcP G134A
Chi²: 0.0391
IL-1RAcP R137A
Chi²: 0.0429
IL-1RAcP Q165A
Chi²: 0.122
IL-1RAcP N166A
Chi²: 0.0952
IL-1RAcP F167A
Chi²: 0.063
IL-1RAcP N168A
Chi²: 0.0679
IL-1RAcP N169A
Chi²: 0.228
IL-1RAcP I171A
Chi²: 0.285
IL-1RAcP E173A
Chi²: 0.0964
IL-1RAcP L180A
Chi²: 0.149
IL-1RAcP L183A
Chi²: 0.0191
IL-1RAcP S185A
Chi²: 0.047
IL-1RAcP H231A
Chi²: 0.116
IL-1RAcP Y249A
Chi²: 0.135
IL-1RAcP S283A
Chi²: 0.0653
IL-1RAcP H284A
Chi²: 0.435
IL-1RAcP R286A
Chi²: 0.0292
IL-1RAcP T287A
Chi²: 0.0599
IL-1RAcP E288A
Chi²: 0.0898
IL-1RAcP E290A
Chi²: 0.0545
IL-1RAcP T291A
Chi²: 0.0344
RU
Time (s)

A **Analyte: CAN03 Fab**

Ligand	*k*a (10^5 M^{-1} S^{-1})	*k*d (10^{-5} S^{-1})	K_D (100pM)
**Wild-type**	3.2 ± 0.6	10.1 ± 3.5	3.1 ± 1.1
**E132A**	2.0 ± 0.5	10.0 ± 0.5	5.1 ± 0.9
**Y133A**	3.5 ± 2.4	10.1 ± 2.5	3.5 ± 1.7
**G134A**	4.3 ± 0.2	13.5 ± 0.0	3.1 ± 0.2
**R137A**	4.3 ± 0.1	11.7 ± 4.3	2.8 ± 1.1
**Q165A**	2.7 ± 0.0	14.9 ± 1.3	5.5 ± 0.5
**N166A**	3.3 ± 0.5	9.5 ± 3.6	2.8 ± 0.7
**F167A**	2.2 ± 0.1	6.4 ± 1.8	2.6 ± 0.7
**N168A**	3.5 ± 0.2	7.6 ± 1.8	2.1 ± 0.4
**N169A**	2.6 ± 0.4	14.7 ± 0.3	5.7 ± 1.1
**I171A**	3.2 ± 0.3	10.4 ± 4.7	3.2 ± 1.2
**E173A**	3.3 ± 0.3	9.4 ± 2.7	2.8 ± 0.6
**L180A**	3.6 ± 0.1	15.7 ± 1.0	4.3 ± 0.2
**L183A**	3.3 ± 0.7	7.4 ± 1.7	2.3 ± 1.0
**S185A**	4.7 ± 0.5	11.5 ± 0.3	2.4 ± 0.3
**H231A**	3.8 ± 0.7	9.0 ± 2.4	2.5 ± 1.1
**Y249A**	2.5 ± 1.3	12.2 ± 1.8	5.3 ± 2.0
**S283A**	3.2 ± 0.5	5.5 ± 0.2	1.7 ± 0.3
**H284A**	3.2 ± 0.1	6.5 ± 1.5	2.0 ± 0.5
**R286A**	4.9 ± 1.4	23.0 ± 3.0	4.7 ± 2.8
**T287A**	3.1 ± 1.1	7.1 ± 1.5	2.5 ± 1.4
**E288A**	3.2 ± 0.0	2.4 ± 0.2	0.8 ± 0.1
**E290A**	3.8 ± 0.6	6.7 ± 1.8	1.7 ± 0.2
**T291A**	3.5 ± 0.4	8.6 ± 2.7	2.5 ± 1.0

B **Analyte: CAN04 Fab**

Ligand	*ka* (10^5 M^{-1} S^{-1})			*kd* (10^{-5} S^{-1})			K_D (100pM)		
**Wild-type**	5.6	±	2.0	9.8	±	5.0	1.8	±	0.6
**E132A**	5.3	±	0.6	18.7	±	6.2	3.6	±	1.6
**Y133A**	4.0	±	0.3	6.5	±	2.3	1.6	±	0.5
**G134A**	4.5	±	0.7	12.1	±	0.4	2.7	±	0.4
**R137A**	5.4	±	1.6	13.1	±	6.8	2.3	±	0.5
**Q165A**	3.0	±	2.2	53.2	±	21.5	20.4	±	7.6
**N166A**	4.1	±	0.4	15.3	±	1.2	3.7	±	0.0
**F167A**	7.3	±	1.1	6.0	±	0.5	0.8	±	0.1
**N168A**	4.3	±	0.2	6.3	±	2.4	1.5	±	0.5
**N169A**	4.1	±	0.1	17.7	±	0.6	4.3	±	0.0
**I171A**	4.7	±	0.2	4.3	±	0.1	0.9	±	0.1
**E173A**	3.8	±	0.1	23.9	±	2.3	6.3	±	0.4
**L180A**	3.3	±	0.4	24.0	±	9.3	7.4	±	3.7
**L183A**	4.5	±	0.2	9.1	±	2.2	2.4	±	0.6
**S185A**	4.6	±	0.1	12.3	±	2.9	2.7	±	0.6
**H231A**	5.7	±	1.5	8.4	±	2.3	1.6	±	0.8
**Y249A**	5.2	±	1.7	9.6	±	2.8	2.0	±	1.2
**S283A**	5.1	±	0.3	9.1	±	1.8	2.0	±	0.3
**H284A**	5.5	±	1.9	8.9	±	1.0	1.8	±	0.8
**R286A**	11.6	±	1.5	7.7	±	0.9	1.3	±	0.5
**T287A**	5.2	±	0.1	5.1	±	1.8	1.0	±	0.4
**E288A**	3.5	±	0.4	10.8	±	2.1	3.1	±	1.0
**E290A**	6.7	±	1.8	8.4	±	3.6	1.2	±	0.2
**T291A**	4.5	±	0.3	10.0	±	0.6	2.2	±	0.0

A

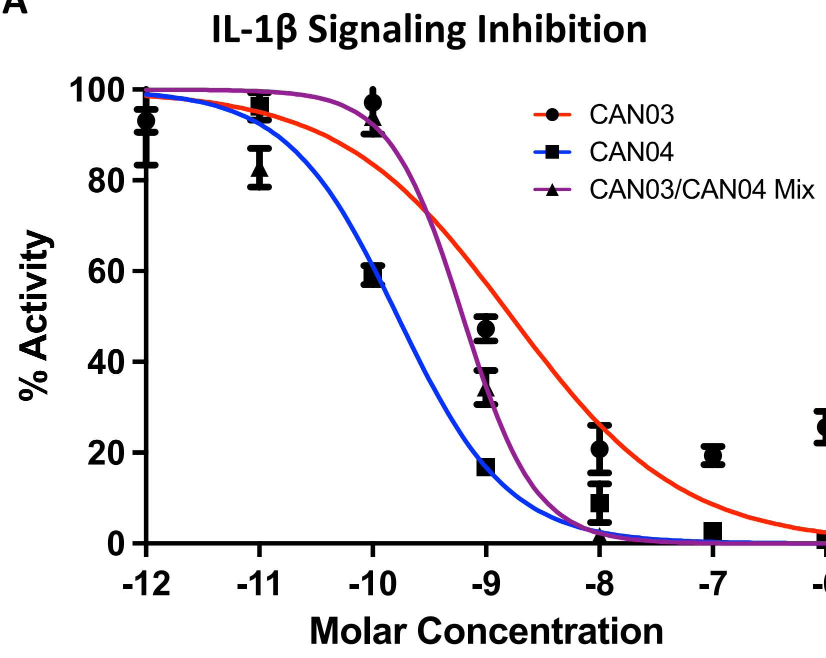
IL-1β Signaling Inhibition
% Activity
100
80
60
40
20
0
-12
-11
-10
-9
-8
-7
-6
Molar Concentration
CAN03
CAN04
CAN03/CAN04 Mix
